# Higher order mode supercontinuum generation in tantalum pentoxide (Ta_2_O_5_) channel waveguide

**DOI:** 10.1038/s41598-021-86922-8

**Published:** 2021-04-12

**Authors:** Ranran Fan, Yuan-Yao Lin, Lin Chang, Andreas Boes, John Bowers, Jia-Wei Liu, Chao-Hong Lin, Te-Keng Wang, Junpeng Qiao, Hao-Chung Kuo, Gong-Ru Lin, Min-Hsiung Shih, Yung-Jr Hung, Yi-Jen Chiu, Chao-Kuei Lee

**Affiliations:** 1 Department of Photonics, National Sun Yat-Sen University, 70, Lienhei Road, Kaohsiung, Taiwan; 2 Center of Crystal Research, National Sun Yat-Sen University, 70, Lienhei Road, Kaohsiung, Taiwan; 3 Department of Electrical and Computer Engineering, University of California, Santa Barbara (UCSB), Santa Barbara, CA 93106 USA; 4 School of Engineering, RMIT University, Melbourne, VIC 3000 Australia; 5 Department of Photonics, National Chiao Tung University, Ta Hsueh Road, Hsinchu 300, 1001 Taiwan; 6 Graduate Institute of Photonics and Optoelectronics, and Department of Electrical Engineering, National Taiwan University, No.1, Sec. 4, Roosevelt Road, Taipei, 106 Taiwan; 7 Research Center for Applied Sciences, Academia Sinica, 128, Sec. 2, Academic Road, Taipei, Taiwan

**Keywords:** Supercontinuum generation, Integrated optics, Supercontinuum generation

## Abstract

We fabricated tantalum pentoxide (Ta_2_O_5_) channel waveguides and used them to experimentally demonstrate higher-order mode supercontinuum (SC) generation. The Ta_2_O_5_ waveguide has a high nonlinear refractive index which was in an order magnitude of 10^–14^ cm^2^/W and was designed to be anomalously dispersive at the pumping wavelength. To the best of our knowledge, this is the first time a higher-order mode femtosecond pump based broadband SC has been measured from a nonlinear waveguide using the phase-matching method. This enabled us to demonstrate a SC spectrum spanning from 842 to 1462 nm (at − 30 dB), which corresponds to 0.83 octaves, when using the TM_10_ waveguide mode. When using the TE_10_ mode, the SC bandwidth is slightly reduced for the same excitation peak power. In addition, we theoretically estimated and discussed the possibility of using the broadband higher-order modes emitted from the Ta_2_O_5_ waveguide for trapping nanoparticles. Hence, we believe that demonstrated Ta_2_O_5_ waveguide are a promising broadband light source for optical applications such as frequency metrology, Raman spectroscopy, molecular spectroscopy and optical coherence tomography.

## Introduction

Supercontinuum (SC) generation is a very attractive third-order nonlinear optical process that can be used to broaden the spectral bandwidth of laser pulses to generate large spectral span^1–3^. SC generation has found widespread use in frequency metrology^4^, Raman spectroscopy^5^, molecular spectroscopy^6^, and optical coherence tomography^7^. For example, with broadband coherent anti-Stokes Raman scattering spectroscopy (CARS) using SC light source the stimulated process of anti-stoke scattering from the molecular vibration/rotation can be efficiently excited and it serves as a fingerprint to identify particular molecular structure^5^.

Advanced SC light sources ranging from ultraviolet to infrared regimes are commercially available by using nonlinear photonic crystal fibers pumped by ns or ps pulse width of laser^8^ which reveals the nonlinear process of modulation instability, soliton interactions, Raman red-shift, self-steepenings^9^. Recently the advancement of micro-fabrication techniques has enabled the fabrication of high-quality and low-loss waveguide devices for photonic integration circuits. On-chip SC light source with the femtosecond laser pump then becomes one of the foci in the photonic society. The broadening range and spectral shape of femtosecond pulse pumped SC generation are mainly determined by the phase-matching condition, input pulse parameters, the nonlinearity of the material, the refractive index and dispersion of the waveguide. When some spectral components generated near the soliton peak are dispersed and resonant with the soliton tails, they are significantly enhanced. These spectral peaks are often referred to as dispersive waves^10–12^.

On-chip nonlinear waveguide platforms offer a lower cost, smaller size, and increased control of the SC light source compared to traditional photonic crystal fibers and bulk crystals. Recently, on-chip SC generation has been investigated in several chip-based systems, such as As_2_Se_3_ chalcogenide glass waveguides^13^, gold nanofilm^14^, silicon photonic nanowires^15^, silicon nitride waveguides^16–18^, silicon–germanium waveguides^19^, silicon waveguides^20–22^, and aluminum nitride waveguides^23^.

Silicon and silicon nitride channel waveguides are very attractive as high purity thin films are commercially available and the patterning processes of silicon and silicon nitride are very mature, thanks to decades of developments of CMOS fabrication processes. Using these techniques enabled the experimental demonstration of octave-spanning SC ranging from 2 to 5 μm^24^ and a frequency comb ranging from 1.5 to 3.2 μm^25^ using a standard silicon channel waveguide. In addition, it is reported that an SC spectrum over 1.5 octaves covering from 2.0 to 5.6 μm with a silicon-on-sapphire platform has been achieved^26^. However, silicon and silicon nitride waveguides suffer from a low SC generation efficiency, as the peak pumping power is limited by the two-photon absorption effect in the core material of silicon/silicon nitride^27–30^. Moreover, a light source that can be integrated with complementary metal-oxide semiconductor CMOS-compatible components will be the key factor for future practical applications^31–34^. To overcome these restrictions, in this study, we proposed and demonstrated a CMOS-compatible channel waveguide for generating SC from the visible to near-infrared range that uses tantalum pentoxide (Ta_2_O_5_) as a waveguide core material. Recently, Ta_2_O_5_ has been utilized to demonstrate waveguide devices, such as ultra-low propagation loss waveguide and vapor sensing applications^35,36^. Furthermore, Ta_2_O_5_ has a very high nonlinear refractive index, which is characterized to be in the order of 10^–14^ cm^2^/W by self-phase modulation (SPM)^37^, four wave mixing^38^ and Z-scan method^39^. It is at least one magnitude higher than that of silica and silicon nitride. The remarkably large third-order nonlinear susceptibility can enable efficient SC generation induced by self-phase modulations and self-steepening. Further, Ta_2_O_5_ has wide band gap of 4.2 eV (corresponding to the photon energy at the wavelength of 295 nm), which reduces the two-photon absorption effect and making it a very attractive material to generate the visible-to-near-infrared SC generation. Ta_2_O_5_ has also a small extinction coefficient, which makes it attractive to guide the SC. Another attractive material property of Ta_2_O_5_ is the small thermal-optical coefficient (2.3 × 10^−6^ K^−1^)^40^, which helps stabilize the performance of waveguide devices under high optical power operations^41^.

Recently, the SC generation community also explored structured illumination to select the SC intensity profile from waveguide devices, which is particularly useful for modern communication systems^42^. Notably SC generation in high order mode fiber modes were demonstrated in nonlinear photonic crystal fibers^43,44^.

However, it is difficult to achieve modal control for a light source with an extremely large spectral extent^45^. In 2018, Hickstein and coworkers measured the SC generated from a guided TE_20_ mode through quasi phase matching via periodic modulations of the waveguide structure in a Si_3_N_4_ waveguide. The quasi phase matching waveguide structure can overcome the difficulty in strong modal dispersion of waveguides^12^.

In this study, we experimentally demonstrate a SC generated in Ta_2_O_5_ channel waveguides by using higher-order modes, which are a phase-matched and do not rely on a sophisticated waveguide quasi-phase matching structure. The waveguide dimensions were chosen to achieve anomalous dispersion for higher-order modes, when using a thermally oxidized Si substrate and a SiO_2_ as the upper cladding. The Oxide cladding provides integration reliability and further distinguished this work from our air-cladding structures published in 2019 in which SC generation extending a spectral range from 565 to 1464 nm is reported^46^. The generation of a guided dispersive wave, which matches the pump soliton index enhances particular spectral intensity in the supercontinuum and broadens its spectral range. The range of SC generation extends from 842 to 1462 nm in the near-infrared (NIR) wavelength for the TM_10_ mode (at − 30 dB), comprising a spectral bandwidth of more than 0.83 octaves. Meanwhile, the TE_10_ mode was also measured, showing relatively smaller SC spectral expansion of more than 0.78 octaves. To the best of our knowledge, this is the first demonstration of higher-order mode femtosecond pulse pumped SC generation extending in a CMOS-compatible nonlinear Ta_2_O_5_ channel waveguide with an anomalously dispersive design.

## Results

**Figure 1:**
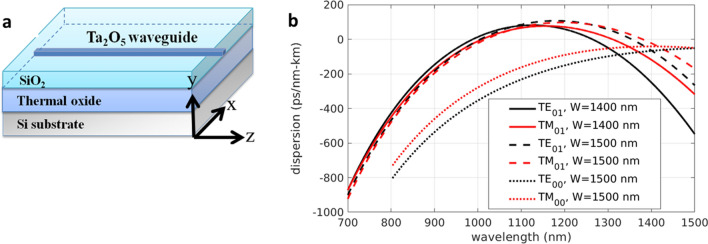
(**a**) Schematic of the structure of Ta_2_O_5_ waveguide. (**b**) Calculated GVD for the higher-order modes of TE (x-) and TM (y-) polarizations in the Ta_2_O_5_ waveguides of 1400 nm and 1500 nm widths.

The SC generated through soliton fission requires waveguides that operate in a low and relatively flat anomalous dispersion regime when the third-order nonlinear coefficient is positive^1^. In this work, we investigate Ta_2_O_5_ channel waveguides as a SC source, as illustrated in Fig. 1a. We determined the thickness (y-) and width (x-) of the channel waveguide on top of the thermal oxide, which is cladded by SiO_2_, by calculating the group velocity dispersion (GVD). The calculated GVDs for the TE (x-) and TM (y-) polarizations for 1400 nm and 1500 nm wide and 850 nm thick waveguides are shown in Fig. 1b. Although according to our calculation, increasing the thickness and width of the waveguide device moves the GVD curve more anomalous and red-shifted, the greater film thickness the more probable cracks during the deposition process because of stress. For a comparison, anomalously dispersed (D > 0) TE_10_ and TM_10_ modes are shown together with the normally dispersed fundamental TE_00_ and TM_00_ modes. The values of GVD at pump wavelength of 1056 nm for the TE_10_ and TM_10_ modes are 47 ps/km-nm and 31 ps/km-nm, respectively. This means that the optical soliton emerges at this wavelength, and the dispersive wave is generated when the phase-matching condition is achieved between the temporal soliton and the dispersive wave. As both the TE_00_/TM_00_ modes in this waveguide are normally dispersive, ultrafast pulses fail to develop soliton fission that explosively broadens the spectrum^1,47,48^. On the other hand, they undergo self-phase modulation (SPM) and broadening in the temporal domain. Consequently, the spectral broadening of TE_00_/TM_00_ modes is expected to saturate and span a spectrum width of 300 nm spanning from 950 to 1250 nm at the peak power of 300 W according to our numerical simulations using the method to be revealed in method section.

**Figure 2:**
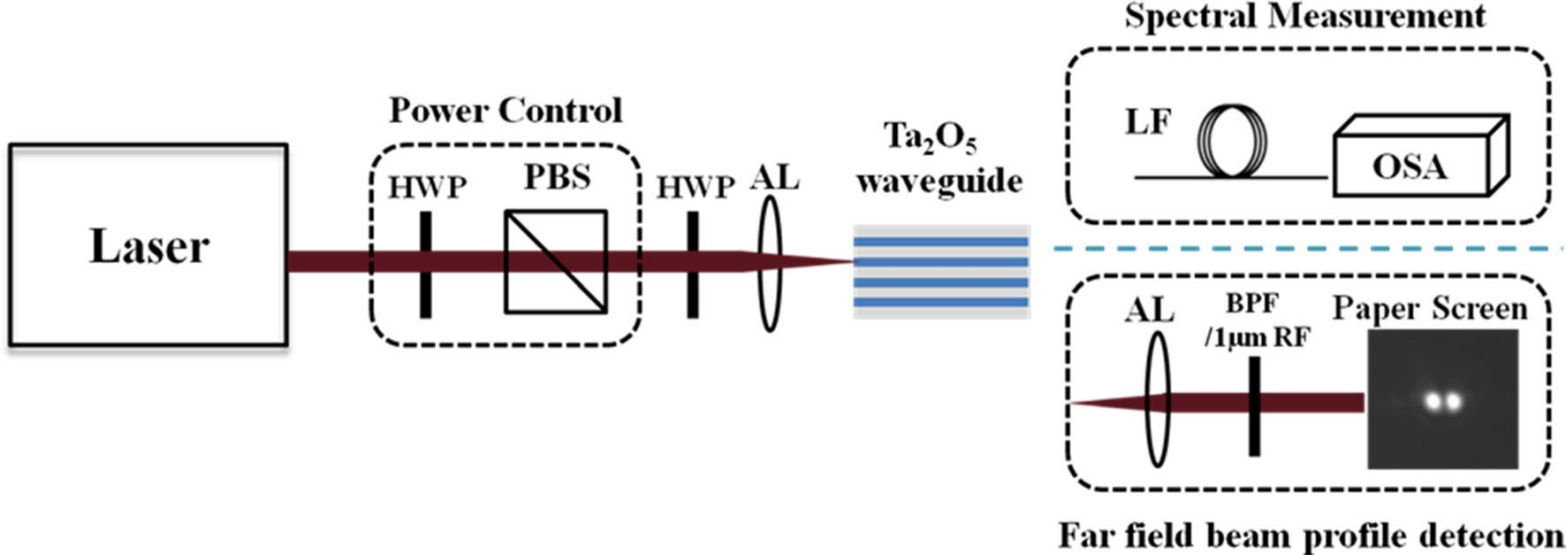
Experimental setup for Ta_2_O_5_ waveguide SC generation. HWP: half-wave plate; PBS: polarizing beam splitter; AL: aspheric objective lens; LF: lensed fiber; OSA: optical spectrum analyzer; BPF: band pass filter; RF: reflector. Laser emits mode-locked pulse at a center wavelength of 1056 nm. The pulse duration is 111 fs and repetition frequency is 18 MHz.

**Figure 3:**
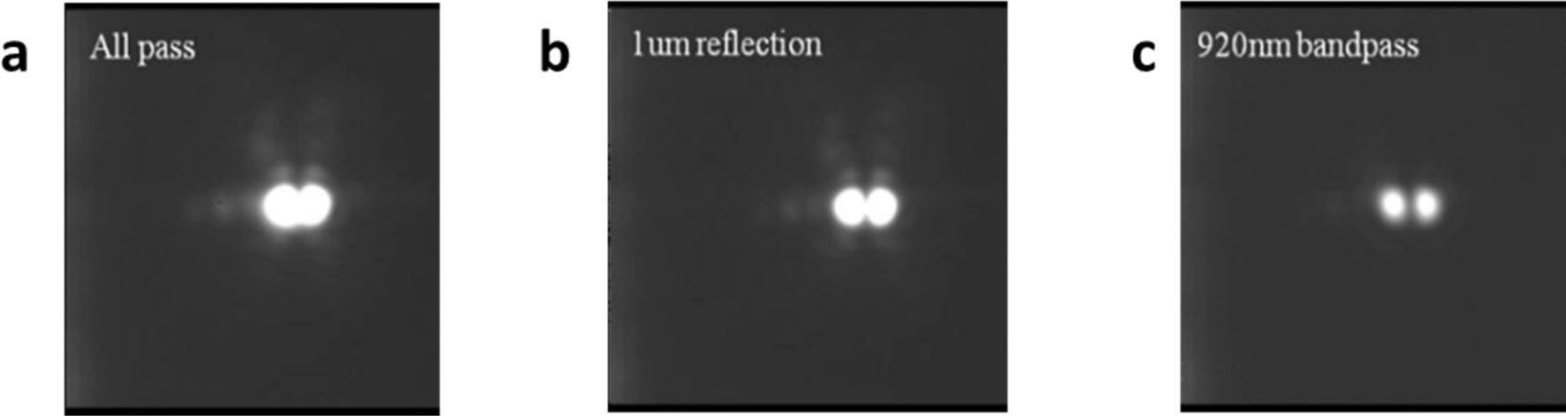
(**a–c**) Distribution of high-order modes without any filter, through a 1 µm reflector and 920 nm band-pass filters.

Experimental setup to generate SC in Ta_2_O_5_ waveguide is shown in Fig. 2. A power control made of a half-wave plate and a polarization beam splitter changes the power of mode-locked laser. A half-wave plate placed after the power control is used to rotate the parallel polarized laser pulse passing through the power control. An aspheric objective lens (100×) is used to couple laser pulse into the Ta_2_O_5_ waveguide. The generated SC is collected by lens fiber and characterized by optical spectrum analyzer. In order to identify the waveguide mode of the SC generation from the experiment system illustrated in Fig. 2, the intensity distribution was recorded by collimating the emitted fields using an aspheric objective lens (100×) and passing through a reflector or a band-pass filter. Finally, the field is first projected onto a paper as a screen for inspection and afterwards directly recorded by a CCD camera. The entire field morphology without a filter is plotted in Fig. 3a, and two ellipses can be seen attached. Next, a 1 µm reflector is placed to suppress the wavelength near the pump, where other bands could pass through it successfully, as shown in Fig. 3b. Two independent circular light fields can be seen, confirming that the mode at the pumping wavelength of 1056 nm should be fundamental, and the SC generation corresponds to higher-order modes. Figure 3c reveals the mode distributions with a 920 nm band-pass filter and the captured mode exposes two distinct light fields that are seen. This confirms that the experiments in this study are operated under the condition of higher-order waveguide modes. Notably the fabricated Ta_2_O_5_ waveguide can support multi-modes. The pump pulse coupled into the waveguide is often mixture of all the possible guided modes.

**Figure 4:**
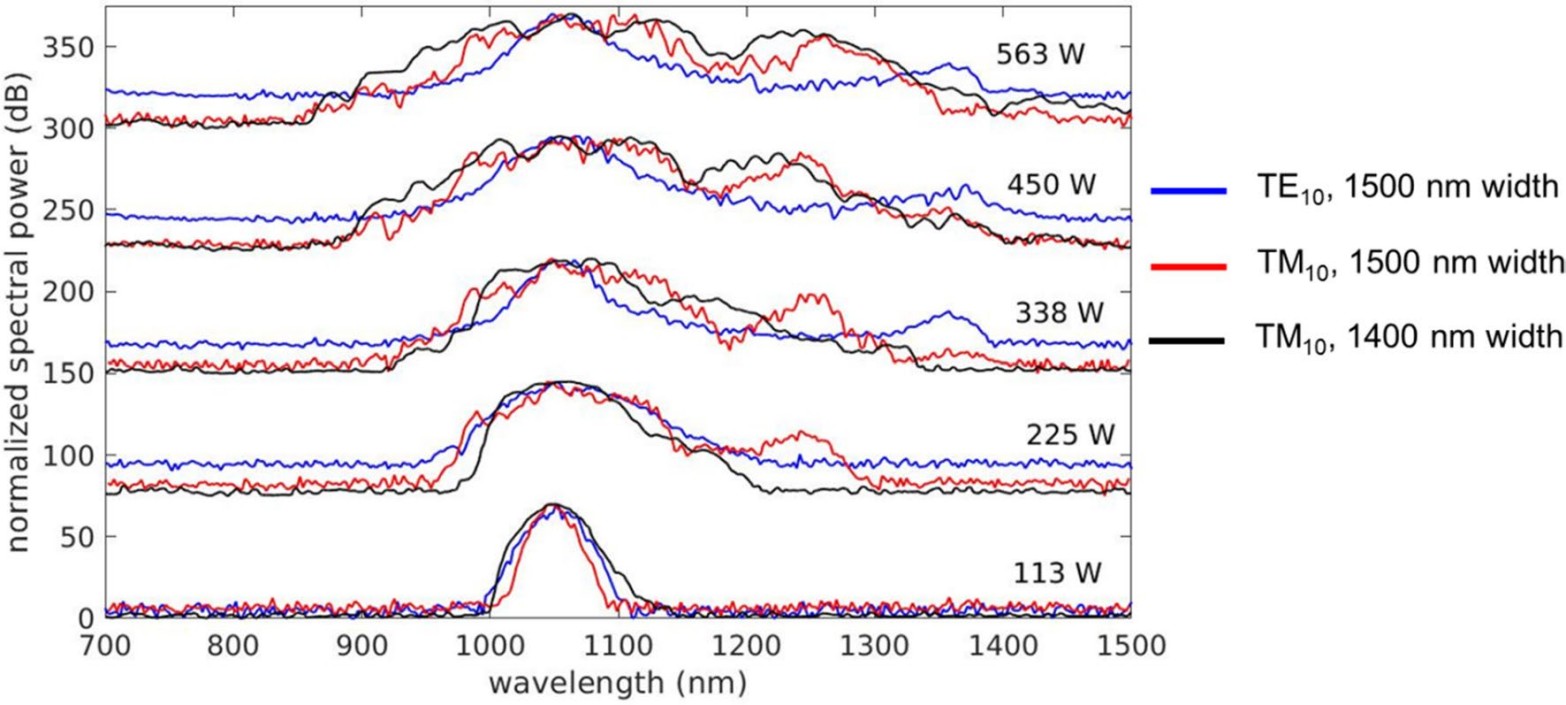
Output spectra of the Ta_2_O_5_ waveguide for the TE_10_ mode (in blue) and TM_10_ mode (in red and in black) with various peak powers coupled to the waveguide. These peak powers estimated by TM_10_ modes are 113 W, 225 W, 338 W, 450 W and 563 W, respectively. The spectra for TE_10_ mode is measured in the waveguide of 1500 nm width (in blue) and the spectra for TM_10_ mode is measured in waveguides of 1500 nm (in red) and 1400 nm (in black) widths.

Afterwards, we analyzed the generated spectrum by coupling the light from the waveguide into a lensed fiber and recorded the spectrum by using an optical spectrum analyzer spanning 700–1500 nm in wavelength. One should be noted that the coupling efficiency from aspheric lens (microscope objective) focused beam in free space to the wave guide is estimated to be − 23.5 dB and propagation loss of the wave guide is less than 1 dB/cm. Figure 4 shows the measured spectra for both the TM_10_ and TE_10_ modes, with various input peak pumping powers coupled to the Ta_2_O_5_ waveguide of 1500 nm width. It can be seen that the spectrum continuously broadens as the peak power increases. For the TM polarization, at a low peak power of 113 W, small ripples emerge at the center of the pulse, suggesting that the self-phase modulation (SPM) effect dominates the form of the spectra^46,49^. At the peak power of 225 W, a peak centered at 1250 nm, which is different from the pumping wavelength, can be seen in the spectrum for the TM_10_ mode. This is attributed to the dispersive wave generation. The generated spectrum (at a − 30 dB level) extends from NIR at 960 nm, and ends at 1280 nm, spanning a total of approximately 320 nm. When the power was increased to a peak level of 338 W, the spectrum spanned 386 nm, starting from 920 to 1306 nm. At a higher peak power of 450 W, SC spectrum spanned 490 nm, from 900 to 1390 nm. At the maximal peak power of 563 W, the spectrum spanned 620 nm from 842 to 1462 nm, achieving more than 0.83 octaves.

When the pump pulses of the same power level were injected, the TE polarized pump induces an SC spectrum inferior to that induced by the TM polarized pump over the entire spectrum. However, it should be noted that the spectral component at the wavelength of 1325 nm emerges when the waveguide is pumped with a peak power of 338 W. This indicates the existence of phase matching to the dispersive wave at 1325 nm for the TE_10_ waveguide mode. As the optical alignment and coupling were optimized for the TM polarization, possible misalignment in the optical pulse upon passing through the half-wave plate after the power controller reduces the coupling efficiency of TE polarizations.

Figure 4 also shows the measured spectra for the TM_10_ mode in the Ta_2_O_5_ waveguide of 1400 nm width. One can see that a 1400 nm wide waveguide is deficient in developing SC generation at low optical powers. This can be explained by the stronger waveguide dispersion for the TM_10_, when compared to the wider waveguide. Thus, the phase matching to the dispersive wave is less efficient. Nevertheless, at high pump power, the SC generation is developed when dispersive waves are successively ejected during soliton fission and higher-order dispersion, leading to fs-level temporal structure^1^. The resonance of dispersive wave radiation and the pump pulse occurs to enhance the dispersive wave radiations that constitute a commensurate SC spectrum to the waveguide of 1500 nm in width.

**Figure 5:**
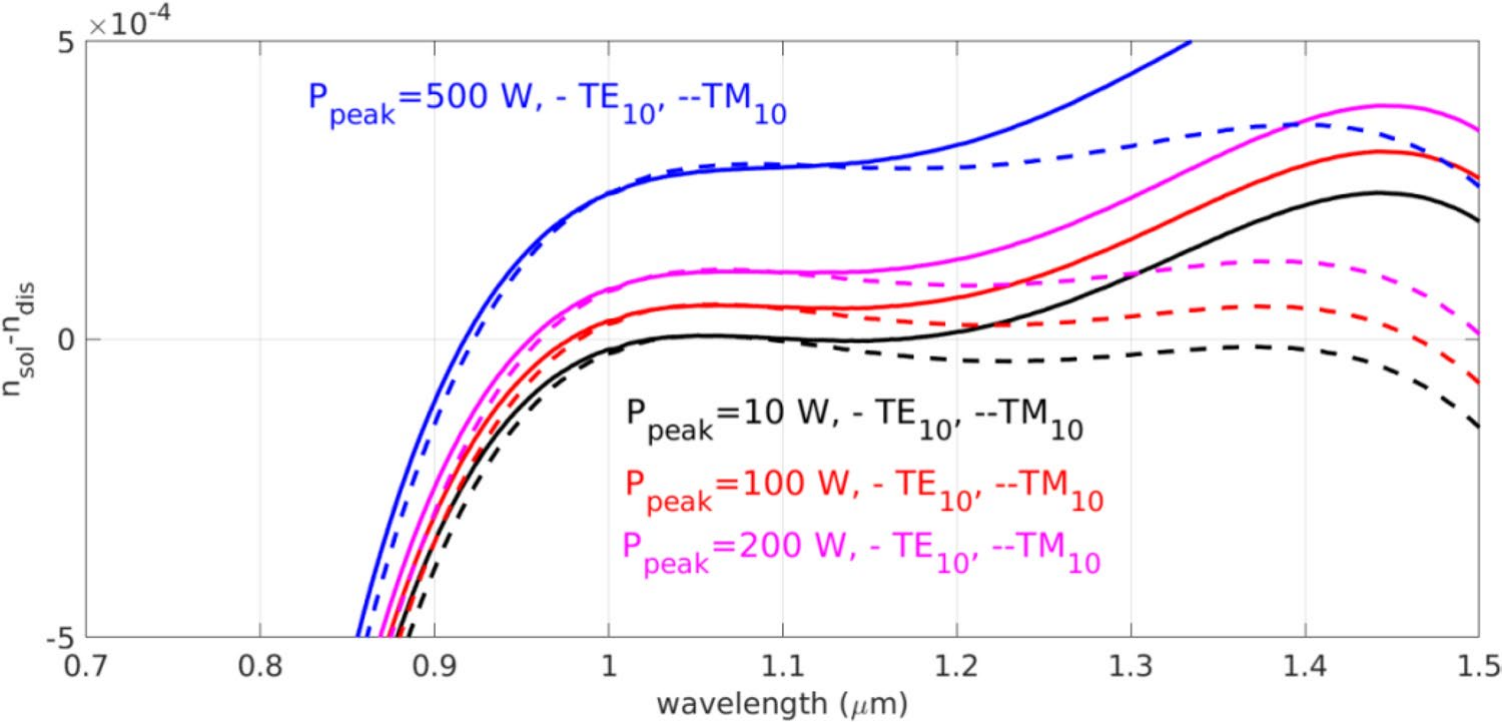
Calculated phase matching condition as the difference between the soliton index and dispersive wave of TE_10_ and TM_10_ modes in waveguide of 1500 nm width under the peak power of 10 W, 100 W, 200 W and 500 W. The nonlinear refractive index of 10^−14^ cm^−2^/W is adopted. The phase matching wavelengths for the dispersive waves of TM_10_ modes in waveguide of 1500 nm width fall in 1.02 μm and 1.1 μm at peak power of 10 W. At the peak power of 100 W, the dispersive waves are at 0.98 μm and 1.46 μm. At the peak power of 200 W, the dispersive waves are at 0.96 μm and 1.5 μm. For higher power, the phase matching falls outsides the experimentally observable range due to the limit of our spectral meter.

According to the calculated dispersion relations, the phase matched dispersive waves can be determined by the curves in Fig. 5, which shows the difference between the power dependent soliton index and dispersive wave index following Eq. (2) in REF 12. At the shorter wavelength region, the wavelength of the phase matched dispersive wave decreased from above 1000 nm to nearly 920 nm as the pump power increases. At the longer wavelength region, the wavelength of the phase matched dispersive wave is 1100 nm at low pump power. As the pump power increases the phase matching dispersive wavelength increases fast due to the large nonlinear coefficient in Ta_2_O_5_. It goes above 1500 nm at the pump power of 200 W for TM_10_ mode. For TE_10_ modes the phase matched wavelength is too far apart from the pump wavelength. Thus, it has weaker spectral component from self-phase modulated pump pulse to resonant with the dispersive wave.

## Discussion

We experimentally demonstrated SC generation at high order waveguide mode but our laser pulse focused by microscope objective is in fundamental Gaussian mode in free space. Through edge coupling approach in free space, most power in the waveguide should propagate in fundamental mode of either polarization, depending on the incident polarization due to the symmetry in the modes. The anti-symmetric mode, such as TE_10_/TM_10_ modes cannot be excited when the incident beam is symmetric. If intense ultrafast pulse coupled to nonlinear waveguide is solely in fundamental mode. The orthogonality of eigenmodes forbid high-order mode to generate by linear coupling. Nonlinear four-wave mixing process in nonlinear waveguide may provide a possible route but it requires phase matching between the two modes. Such an environment is not always guaranteed in nonlinear wave propagation unless it is designed intentionally.

**Figure 6:**
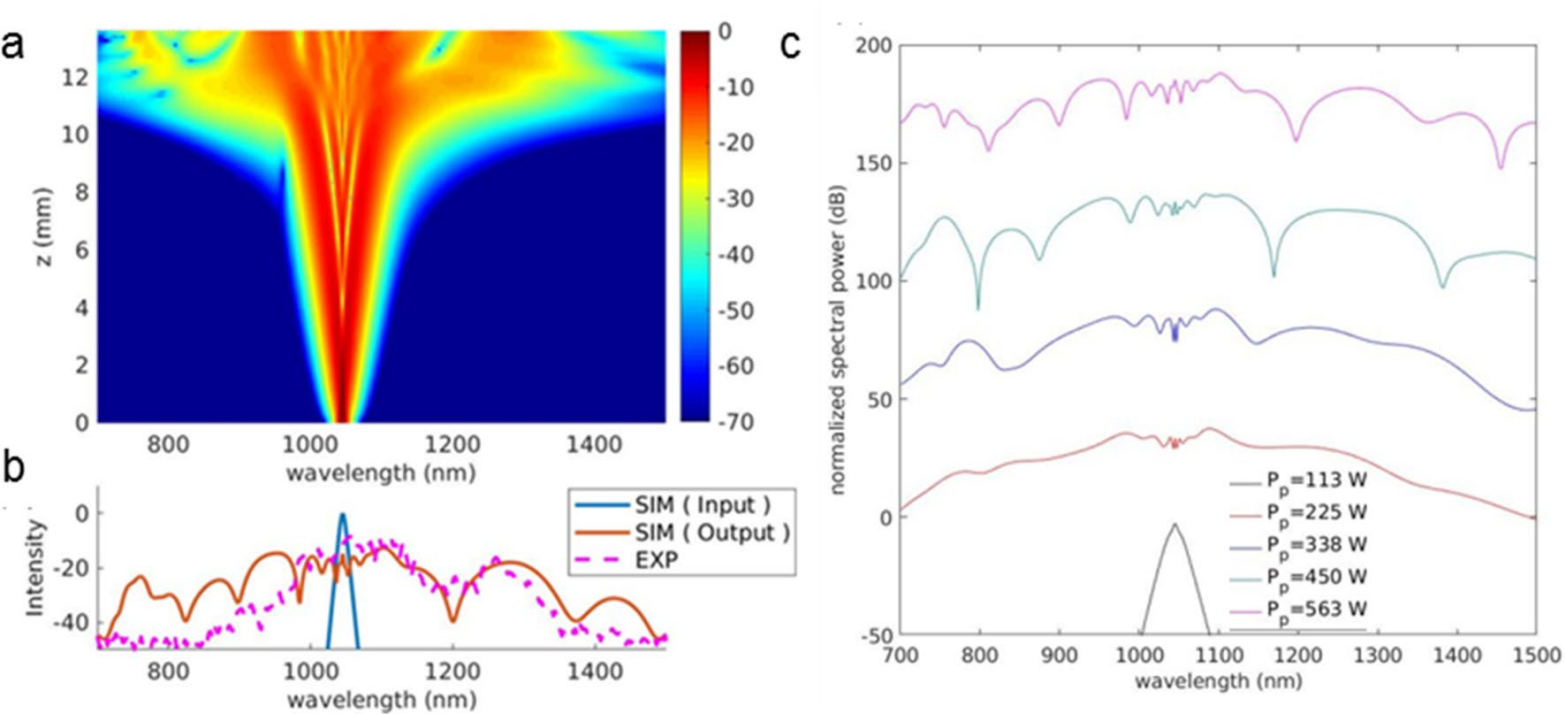
(**a**) Simulated SCG of TM_10_ mode at the peak power of 563 W coupled into the Ta_2_O_5_ waveguide: (**a**) spectral intensity; (**b**) comparison of the simulated (in red) and measured (in magenta) spectra at the output. The spectrum of the input pulse is shown in blue. (**c**) Estimated SC spectrum generated with ultrafast pulses of various peak powers coupled to the Ta_2_O_5_ waveguide.

The second scenario is that both fundamental and high order waveguide modes are coupled into the waveguide simultaneously under misaligned fundamental pump. The misalignment of incident laser breaks the symmetry of the incident laser and the anti-symmetric TE_10_/TM_10_ can be excited along with fundamental modes. Usually, the beam radius of laser beam focused by microscope objective is much larger than the waveguide dimension. When the pump is misaligned by a small angle, the coupling efficiency of fundamental slightly decreases and the coupling efficiency of high order waveguide mode increases almost linearly proportion to the misaligned angle. More than 0.2% coupling efficiency can be achieved for high order waveguide mode with a misaligned angle of 5 degrees (shown in Fig. 6 below). With a − 23 dB coupling efficiency from focused laser pulse to waveguide, roughly averaged power of 20 mW is coupled into the waveguide, the portion of high order waveguide mode could be as large as 2.2 mW. It is comparable to simulation result using single mode input. Notably excitation of pure high order guided mode in nonlinear waveguide is always difficult without an insertion mode converting devices and to distinguish portion of modes is also difficult experimentally.

To better elucidate the SC generation process assisted by phase-matching the dispersive wave at higher-order modes (TM_10_) in the Ta_2_O_5_ waveguide, we consider a widely adopted model of Nonlinear Schrödinger Equation (NLSE)^48^. Using the NLSE evolution routine (see Methods for details), the spectral broadening during propagation is shown in Fig. 6a. In the propagation region below 5 mm, the spectrum broadening is mainly due to SPM, which exhibits an almost liner broadening with saturation effect. An abrupt spectral expansion occurs at the location above 7 mm where the high order dispersion and the accumulated nonlinear phase break up the pulse in temporal domain into a train of solitons^50^. It offers rapid intensity variation, and large nonlinear phase modulation is induced to produce new frequency components and thus most of the SCG bandwidth is obtained after propagation of approximately 8 mm. The spectral width and the positions of the dispersive wave in the simulation and experiment are shown in Fig. 6b. The calculated spectrum reveals a dispersive wave emerging with peaks around 960 nm and 1250 nm in the shorter and longer wavelength regime, respectively, as shown in Fig. 6b. The shape of the estimated output spectrum demonstrates a reasonable agreement with the measurement results in the longer wavelength regime. In the shorter wavelength regime, the SC spectrum in the experiment is deficient and the dispersive wave in the shorter wavelength regime is not clear. We believe that the even stronger dispersion in the shorter wavelength region weakens the phase matching to the dispersive waves. It is worth to emphasize that as shown in Fig. 5 the strong dispersion at shorter wavelength limits the bandwidth of dispersive wave and the spectral broadening in shorter wavelength region accordingly. Although the discrepancy exists between the calculation and experiment, their power dependent spectral response is commensurate. Moreover, higher-order dispersions might play important roles in the formation of the SC spectrum. From Fig. 6b, one can see that the estimated SC spectrum extends wider for longer wavelengths than that for the short wavelengths. Although higher-order dispersions are essential to initiate the SC generation process, they also determine the phase-matching bandwidth between the solitary pump and the dispersive waves. They could restrain the SC bandwidth if they are very large. The pump pulse quickly expands in time as propagation progresses, and the temporal optical intensity is therefore reduced. Consequently, the nonlinear interaction near the dispersive band must be weakened.

Moreover, it is instructive to look into the power evolution of the TM_10_ mode in the Ta_2_O_5_ nonlinear waveguide. Figure 6c plots the spectral intensity with varying peak power continuously from 113 to 563 W. Notably, the estimated spectrum is similar to the experimental results shown in Fig. 4. At the peak power of 225 W, the dispersive wave emerges at approximately 1250 nm in the infrared wavelength, which greatly extends the width of the SC spectrum. At the peak power of 338 W, the estimated spectrum shows that a dispersive wave might emerge at a wavelength of 800 nm. At the peak power of 563 W, a dispersive wave with a peak at about 1400 nm in the infrared region emerges. It should be noted that the fully developed SC spectrum measured in the experiment is similar to the simulated SC spectrum only in the infrared regime even though a dispersive wave may appear in the visible regime according to the calculated dispersion relation. However, the variation of anomalous dispersion in the visible region is more rapid than that in the infrared region, and the dispersive wave generated in the visible range should be less efficient, as is also confirmed experimentally in Fig. 4.

In general, using an air cladding scheme for generating octave-spanning SC is a common method and has been employed for various nonlinear waveguide materials^26,46,49^. However, robustness and stability issues are inevitable. Here, the oxide cladding structure reveals the reliability of the waveguide and is essential for practical application such as frequency combs^17,18^. Moreover, despite the anomalous dispersion for fulfilling phase matching within the SC generation process being crucial, efficient SC generation is still based on the nature of large χ^(3)^. The SC generation from the higher-order mode also exhibits high optical nonlinearity in Ta_2_O_5_, even though the peak intensity of the pump pulse is reduced due to the larger effective area of the higher-order mode. Additionally, from Fig. 3a,b,c, it is clear that the broadband TE_10_/TM_10_ mode can be realized.

Finally we proposed and discussed the possible application of higher-order mode SC for efficient trapping of nanoparticles. Till date, SC has shown its potential for wide range of applications, such as metrology, and OCT. In particular, the coherent property of the SC generation process with a very large spectral width has made the SC light source applicable in performing spectroscopy and particle manipulation simultaneously, which is known as SC tweezer^5,51^. Typically, for trapped nanospheres, the scattered spectrum can be analyzed for investigating the nanosphere for material studies. In addition to trapping particles using a single tightly focused beam trap or dual-beam trap^52^, higher-order laser modes such as Laguerre-Gaussian for efficient transverse trapping have been demonstrated^53^, which did low damage to some vulnerable biomedical samples. Furthermore, three-dimensional optically trapped structures as a superposition of higher-order modes were proposed for optically manipulating an array of particles^54^. Using the high order waveguide mode SC light from the Ta_2_O_5_ waveguides, the forces acting on a nanosphere, by considering scattering in the Rayleigh limit offers the opportunity to trap particles that can only sustain low optical intensities (See methods for details). As a result, the large bandwidth in the wave packet generated from our TE_01_/TM_01_ guided wave can potentially be applied for future compact SC optical tweezers.

## Conclusion

In conclusion, we demonstrated Ta_2_O_5_ channel waveguides, which was deposited by e-beam evaporated, patterned and cladded with a SiO_2_ layer by PECVD. The waveguides have a high nonlinear refractive index and the waveguide dimensions were designed to achieve anomalous dispersion for higher-order modes at the pumping wavelength and to realize the phase-matching. The measured SC spectra span 0.83 octaves (at − 30 dB) wide for the TM_10_. To the best of our knowledge, this is the first demonstration of higher-order mode SC extending in a nonlinear waveguide using a direct phase-matching method. This integrated platform offers an energy-efficient, CMOS compatible, and visible to a near-infrared broadband source, which will be an excellent candidate for frequency metrology, Raman spectroscopy, and optical coherence tomography. We also proposed and discussed the possible application of higher-order mode SC for efficient trapping of nanoparticles.

## Methods

### Waveguide devices fabrication

A 840 nm thick Ta_2_O_5_ thin film was deposited on the thermally oxidized Si wafer by e-beam evaporation, accompanied by ion-assisted treatment^39^. Afterwards, the Ta_2_O_5_ film was annealed in the O_2_ environment at 580 °C in order to compensate for the oxygen deficiency generated during the evaporation process. Afterwards, waveguides with widths of 1400 nm and 1500 nm were fabricated in the film using an e-beam lithography process and a dry etching method. The length of the waveguide was 13.2 mm. Lastly, the Ta_2_O_5_ waveguide was cladded with a SiO_2_ layer with a thickness of 2 µm by plasma-enhanced chemical vapor deposition (PECVD).

### The optical setup for characterizing the supercontinuum generation of the fabricated Ta_2_O_5_ channel waveguides

In the experiment SC generation system is illustrated in Fig. 2. The waveguides were optically pumped with ultrafast pulses from a mode locked laser with a central wavelength of 1056 nm, pulse duration of 111 fs and repetition rate of 18 MHz. A power controller composed of a half-wave plate and a polarizing beam splitter, and an additional half-wave plate were installed to enable the independent control of power and polarization of the laser pulses. The pump was coupled to the waveguide by a 100× microscope objective. The coupling loss at the input was approximately 23.5 dB, caused by the mode mismatch. The maximal power coupled into the waveguide at high order waveguide mode was approximately 2.2 mW, corresponding to a peak pumping power of 563 W.

### Numerical simulation of the supercontinuum generation

**Figure 7:**
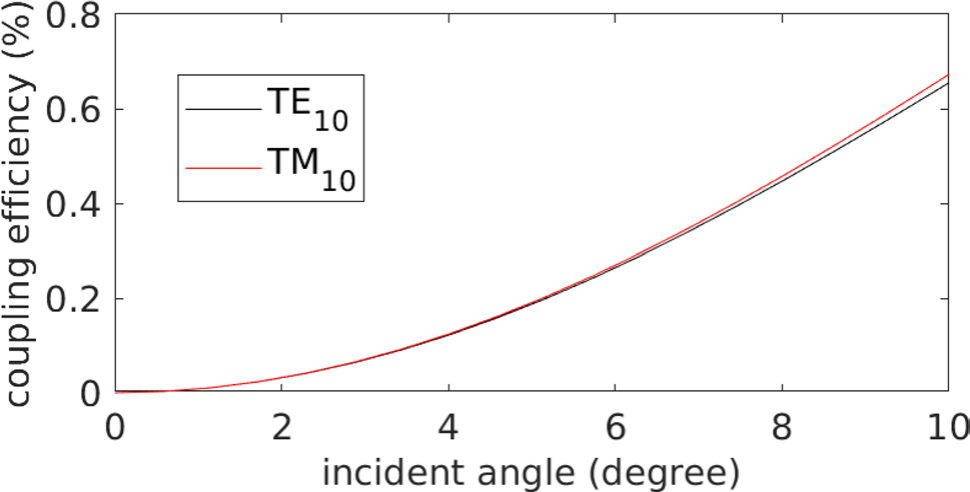
The coupling efficiency of focused beam misaligned at various incident angles.

We consider a widely adopted single mode model of Nonlinear Schrödinger Equation (NLSE)^48^.

1 $$\frac{{\partial A\left( {z,t} \right)}}{\partial z} = \mathop \sum \limits_{n \ge 2} \frac{{i^{n + 1} }}{n!}\beta_{n} \frac{{\partial^{n} A}}{{\partial t^{n} }} + i\gamma \left( {\left| A \right|^{2} A + \frac{i}{{\omega_{0} }}\frac{{\partial \left( {\left| A \right|^{2} A} \right)}}{\partial t}} \right)$$

where *A*(*z*,*t*) is the spatial–temporal complex envelope function, *t* is the retarded time moving at the group velocity of pump pulse of a specific waveguide mode, *z* is the axial propagation distance, *ω*_0_ is the pump frequency, *β*_*n*_ is the *n*th order dispersion, and *γ* is the nonlinear coefficient of the waveguide. This equation describes the temporal evolution of the complex envelope function of the electric field in a nonlinear medium. The third-order nonlinear polarizations, including the Kerr effect and self-steepening, are of particular importance for SC formation. Raman shift is one of the important interaction in fiber based SC generation that is responsible to red shift of the spectrum under long propagation distance. The Raman spectra of amorphous Ta_2_O_5_ thin film is peaked at 200 cm^−1^ and 700 cm^−1^, respectively^55^ and the corresponding wavelengths are 50 μm and 14.28 μm. But the effective nonlinearity of Raman compared to Kerr effect has never been reported. Thus we assumed Raman effects insignificant and it is excluded in the NLSE. It turns out that the simulation results without Raman scattering match the experimental spectrum in long wavelength regime within a short propagation distance about 1 cm. As the ultrafast pulse-coupled into the waveguide is finely tuned subject to the SC spectrum, higher-order mode distributions through a 1 µm reflector or the band-pass filters can be seen in Fig. 3. The experimental observation confirms that the SC is generated at higher-order modes, which experience an anomalous dispersion. Although the formation of SC at higher-order modes could be disputable even in the framework of multimode SC generation^56^, herein, it is assumed in our work that higher-order mode accounts for a considerable portion in the formation of SC. Our laser pulse focused by microscope objective is in fundamental transverse mode. Through edge coupling approach in free space, the anti-symmetric mode, such as TE_10_/TM_10_ modes cannot be excited when the incident beam is symmetric. If intense ultrafast pulse is coupled to nonlinear waveguide with a solely fundamental mode, the orthogonality of eigenmodes forbid the generation of high order waveguide mode through linear coupling. Nevertheless, the effective refractive index of fundamental mode is greater than that of high order waveguide modes in Ta_2_O_5_ waveguide. Large modal dispersion overwhelms the chromatic dispersion and phase matching for nonlinear mode conversion can hardly be achieved. The degenerate four-wave mixing, which has the strongest nonlinear mixing power cannot achieve phase matching from fundamental mode to higher order mode in any combination of high order polarizations shown in the figure below. Such phase mismatch leads the coherent length of the degenerate four-wave mixing to be less than 8 μm. Therefore, one concludes that both fundamental and high order waveguide modes are coupled into the waveguide simultaneously under misaligned Gaussian beam pumping. The misalignment of incident laser breaks the symmetry of the incident laser and the anti-symmetric TE_10_/TM_10_ can be excited along with fundamental modes. Usually, the beam radius of laser beam focused by microscope objective is much larger than the waveguide dimension. When the pump is misaligned by a small angle, the coupling efficiency of fundamental slightly decreases and the coupling efficiency of high order waveguide mode increases almost linearly proportion to the misaligned angle. More than 0.2% coupling efficiency can be achieved for high order waveguide mode with a misaligned angle of 5 degrees as shown in Fig. 7. With a − 23 dB coupling efficiency from focused laser pulse to waveguide, roughly averaged power of 20 mW is coupled into the waveguide, the portion of high order waveguide mode could be as large as 2.2 mW. It is comparable to simulation result using single mode input. Notably excitation of high order guided mode in nonlinear waveguide is always difficult without an insertion mode converting devices and to distinguish portion of modes is also difficult experimentally.

Adopting the calculated dispersion relation of TM_10_ at 1056 nm, which is summarized in Table 1, the generated SC spectrum can be estimated using NLSE normalized to dimensionless coordinates and implemented using a split-step Fourier algorithm, considering higher dispersions up to the fourth order^46,48^, in which the differential operation of 2nd to 4th orders are computed in frequency domain and transform back to time domain. Dispersion coefficients at pump wavelength are obtained from the numerical calculated effective index of TM_10_ modes as shown in Table 1. It is worth mention that the axial extent of the envelope function of the ultrafast pulse of 111 fs is over 30 μm, which is much larger than the transverse dimension in the waveguide. Therefore, the field distribution of the mode and its corresponding dispersion relation are assumed to be invariant during the wave evolution. The waveguide dimensions are restricted to a width of 1500 nm and a height of 840 nm. The Ta_2_O_5_ waveguide length was 13.2 mm. The effective nonlinear coefficient is 6.61 W^−1^ m^−1^, given a mode area of 0.8 µm^2^. The numerical simulation starts with a hyperbolic shaped pulse having a pulse width and amplitude corresponding to that of the pump pulse. In the simulation, 2000 points with temporal increments of 1 fs sample the pulse envelope and the step-size in propagation is 6.6 μm. The nonlinear Kerr effect and self-steepening are considered in the model to investigate the SC generation in an averaged manner. Neither quantum noise nor laser-technical noise is considered due to its relative intensity noise is much lower than the 30 dB limit of the spectral components of interest^50^.

**Table 1:** Dispersion parameters for a waveguide width of 1500 nm and TM_10_ polarization.

Symbol	Name	Value
*β*_2_	Quadratic dispersion	− 0.02933 ps^2^/m
*β*_3_	Cubic dispersion	0.00035 ps^3^/m
*β*_4_	Quartic dispersion	1.7193 × 10^–7^ ps^4^/m
*Γ*	Nonlinear coefficient	6.61 W^−1^ m^−1^

### Numerical simulation of the high order waveguide mode tweezer

**Figure 8:**
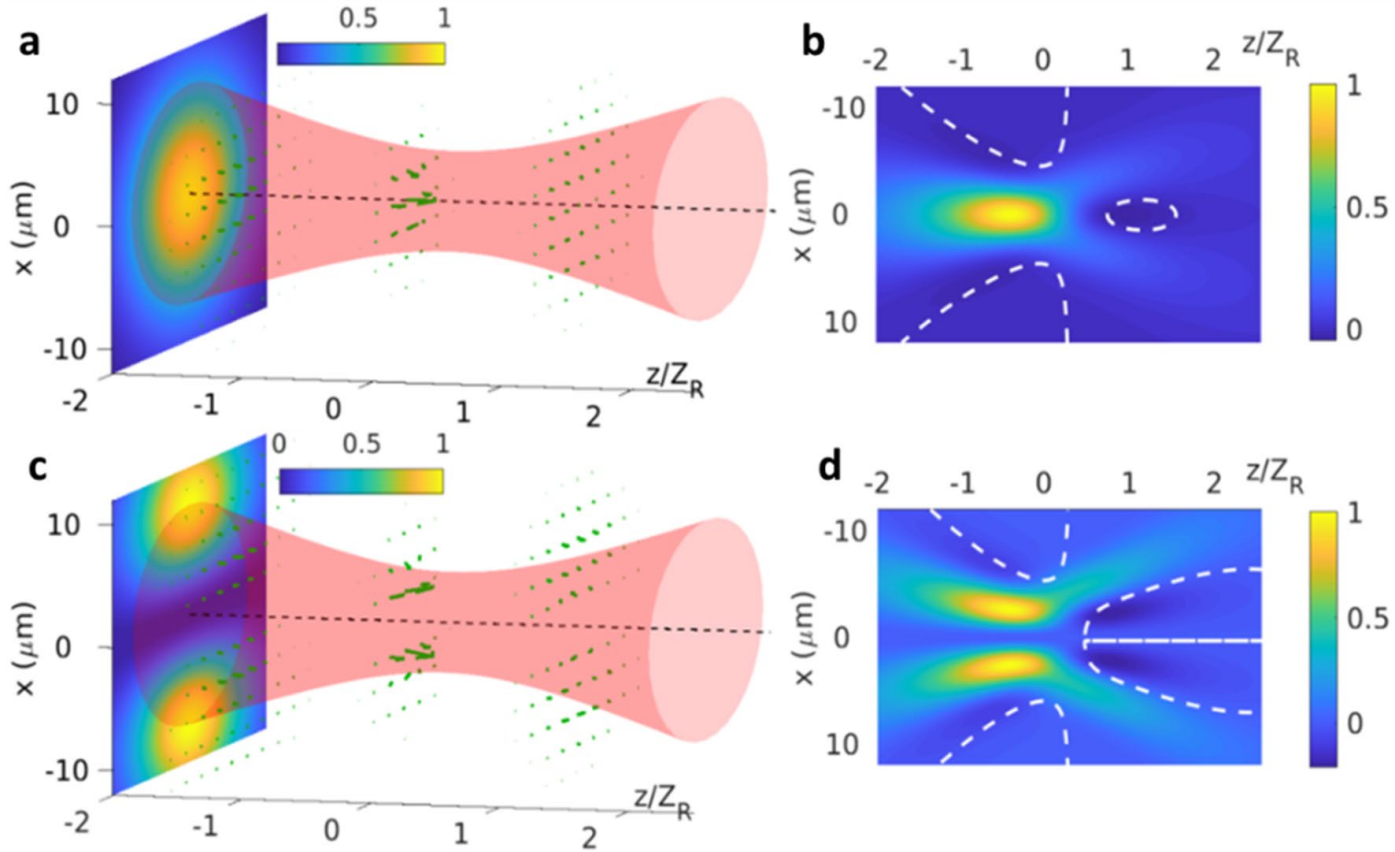
(**a**) Force exerted on a spherical (Rayleigh) particle of 30 nm in diameter when it is illuminated by a tightly focused fundamental Gaussian beam of 4 μm in radius at the focal point. m = 1.097 is used. (**b**) The normalized *z*-component of the radiation force on *y* = 0 plane. The zone of vanishing *z*-component of the radiation force is along the dashed line in white. (**c**) The exerted force on a spherical (Rayleigh) particle of 30 nm in diameter for TE_10_ (TM_10_) mode of the same beam radius. (**d**) The normalized *z*-component of the radiation force on *x* = 0 plane.

To investigate the performance of the generated SC from the Ta_2_O_5_ waveguides, we estimate the forces acting on a nanosphere, by considering scattering in the Rayleigh limit. The forces acting on nanosphere are^48^,

2 $$\vec{F}\left( {\vec{r}} \right) = \vec{F}_{scat} \left( {\vec{r}} \right) + \vec{F}_{grad} \left( {\vec{r}} \right)$$

in which

$$\vec{F}_{scat} \left( {\vec{r}} \right) = \hat{z}\frac{{128\pi^{5} R^{6} }}{{3c\lambda_{0}^{4} }}\left( {\frac{{m^{2} - 1}}{{m^{2} + 2}}} \right)^{2} n_{s}^{5} I\left( {\vec{r}} \right);\quad \vec{F}_{grad} \left( {\vec{r}} \right) = \frac{{2\pi n_{s} R^{3} }}{c}\left( {\frac{{m^{2} - 1}}{{m^{2} + 2}}} \right)\nabla I\left( {\vec{r}} \right)$$

are the scattering and gradient forces from the incident electromagnetic waves, respectively. *m* is the ratio between refractive indices of the nanosphere and its surroundings; *R* is the radius of the nanosphere; *c* is speed of light; *n*_*s*_ is the refractive index of nanoshphere; $\lambda_{0}$ is the laser wavelength; $I\left( {\vec{r}} \right)$ is the intensity distribution of the laser beam. With *m* > 1 Eq. (2) delineates the forces from focused Gaussian beam and TE_10_/TM_10_ modes are qualitatively illustrated in Fig. 8a,c, respectively. With fundamental Gaussian the transverse trapping is achieved by gradient force that pushes the nanosphere to the beam center with of stronger intensity. On the contrary, the TE_10_/TM_10_ mode provides transverse confinement to nanosphere in *x*-direction by balance of the pulling force. As shown in Fig. 8b,d, in *x*-plane the z-component of the forces turns negative after the focal point indicating stable trapping capability in the structure. With the same focusing condition, it is clearly seen in Fig. 8b,d that TE_10_/TM_10_ mode is more robust for trapping nanosphere where the region with negative z-component of force is larger. Near the beam center, the scattering force for TE_10_/TM_10_ is very small and can be easily compensated by gradient force. It offers an opportunity to trapped particles that can sustain low optical intensity. As a result, the large bandwidth in the wave packet generated from our TE_10_/TM_10_ guided wave can potentially be applied for future compact SC optical tweezers.

## Data Availability

The data that support the findings of this study are available from the corresponding authors, Y.-Y. Lin and C.-K. Lee, upon reasonable request.
